# Does volatile sedation with sevoflurane allow spontaneous breathing during prolonged prone positioning in intubated ARDS patients? A retrospective observational feasibility trial

**DOI:** 10.1186/s13613-019-0517-8

**Published:** 2019-03-25

**Authors:** Jascha Heider, Joachim Bansbach, Kai Kaufmann, Sebastian Heinrich, Torsten Loop, Johannes Kalbhenn

**Affiliations:** Department of Anesthesiology and Critical Care, Medical Center – University of Freiburg, Faculty of Medicine, University of Freiburg, Hugstetter Strasse 55, 79106 Freiburg, Germany

**Keywords:** ECMO, ARDS, Prone positioning, Sedation, Spontaneous breathing, Sevoflurane, Volatile anesthesia

## Abstract

**Background:**

Lung-protective ventilation and prolonged prone positioning (PP) are presented as essential in treating acute respiratory distress syndrome (ARDS). The optimal respirator mode, however, remains controversial. Pressure-supported spontaneous breathing (PS) during ARDS provides several advantages, but is difficult to achieve during PP because of respiratory depression as a side effect of sedative drugs. This study was designed to evaluate the feasibility and safety of PS during PP in ARDS patients sedated with inhaled sevoflurane.

**Results:**

Overall, we have observed 4339 h of prone positioning in 62 patients who had a median of four prone episodes during treatment. Within 3948 h (91%), patients were successfully brought into a pressure-supported spontaneous breathing mode. The median duration of each prone episode was 17 h (IQR 3). Median duration of pressure-supported spontaneous breathing per episode was 16 h (IQR 5). Just one self-extubation occurred during 276 episodes of PP.

**Conclusions and implications:**

Pressure-supported spontaneous breathing during prolonged prone positioning in intubated ARDS patients with or without ECMO can be achieved during volatile sedation with sevoflurane. This finding may provide a basis upon which to question the latest dogma in ARDS treatment. Our concept must be further investigated and compared to controlled ventilation with regard to driving pressure, lung-protective parameters, muscle weakness and mortality before it can be routinely applied.

## Background

Adherence to a bundle of respirator settings termed “lung-protective ventilation” [1, 2] and prolonged prone positioning (PP) [3–5] are presented as essential in guidelines for treating patients with acute respiratory distress syndrome (ARDS) [6]. What the optimal respirator mode in ARDS is, however, remains controversial [7]: pressure-supported spontaneous breathing (PS) lowers intrathoracic and driving pressure, improves oxygenation, may partly recruit atelectatic areas of the lung [8, 9], supports the diaphragm preventing lung collapse [10], does not worsen right ventricular function [11, 12] and prevents patients from ICU-acquired muscle weakness [13–17]. The risk of spatial hyperinflation due to increased transpulmonary pressure, ventilator-to-patient-dys-synchrony and the risk of ventilator induced lung injury (VILI) as a consequence [18, 19] on the other hand may support controlled ventilation and even muscle relaxation in early ARDS to achieve “lung protection” [20]. In a large international observational trial, spontaneous breathing was present in 46% of patients with severe ARDS but had no effect on mortality. Spontaneous breathing was associated with more ventilator-free days and a briefer ICU stay [21].

In recent trials, patients were mechanically ventilated in a *controlled* ventilatory mode during PP under deep intravenous sedation [3, 4, 22]. The combination of PP and *supported spontaneous* breathing has not been systemically evaluated so far due to the dilemma between sedation necessary to tolerate PP and sedative-induced depression of respiratory genesis [15, 23, 24]. Sedatives compromise respiratory genesis in a dose-related manner. In contrast to intravenous sedatives; however, an effective sevoflurane concentration is traceable in real time in the exhaled air. Moderate sedation is thus more likely achievable concomitant with spontaneous breathing [25]. It also enhances a faster recovery and shortens time to extubation in ICU patients [26]. Inhaled sevoflurane is known to be safe when applied for a prolonged period during intensive care unit (ICU) therapy [27].

We hypothesized that pressure-supported spontaneous breathing during PP would be feasible during volatile sedation with sevoflurane. In this retrospective observational trial, we evaluated a cohort of patients with moderate or severe ARDS. The primary endpoint was length of PS in hours related to the PP duration. Secondary outcome parameter was the incidence of sedation-related unexpected events.

## Methods

**Fig. 1:**
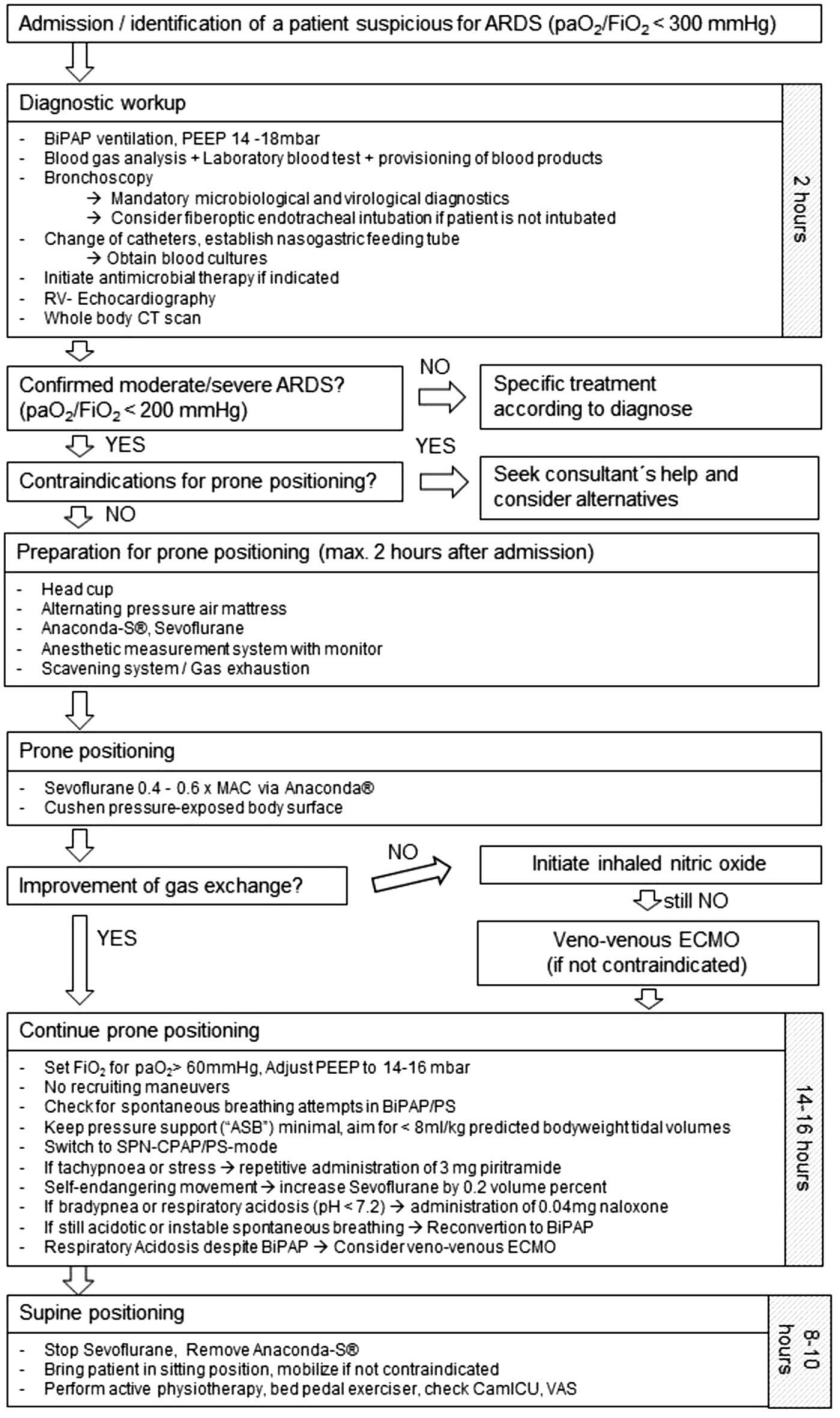
Departmental standard operating procedure for workup in ARDS

Patients were recruited in a German University ARDS center und ECMO center endowed according to international recommendations [28] and certified by the German Society of Anesthesiology and Intensive Care Medicine (DGAI). Patients with ARDS according to the Berlin definition [29] (onset within 1 week; bilateral opacities, not explained by cardiac failure or hypervolemia; paO2/FiO2 < 300 with PEEP ≥ 5 cmH_2_O) were treated according to a standardized departmental protocol (Fig. 1). When computed tomographic findings failed to support ARDS, the diagnosis was rejected. Patient data are anonymously collected in a local ARDS registry. The study was approved by the local ethics committee (EK 141/17) and the general contract governing medical treatment. It was confirmed that no further informed consent was necessary because of the study’s descriptive, non-interventional and anonymous design which was planned and designed in accordance with the initiative for Strengthening the Reporting of Observational Studies in Epidemiology (STROBE), using the suggested checklist for epidemiological cohort studies [30].

### Patients

The local ARDS registry was scanned for all patients treated for at least 24 h with a prescription of sevoflurane since the registry’s introduction in 2014. Patients not at least 18 years old were excluded. Patients not suitable for PP (e.g., external pelvic fixateur, spine injury, abdomen apertum, etc.) or those who only revealed side positioning or incomplete PP (130°) were also excluded from analysis.

### Analgosedation

Sedation was established via inhaled sevoflurane (Sevoflurane Baxter, Baxter Germany GmbH, Unterschleissheim, Germany) applied continuously with the AnaConDa™ System (Sedana Medical AB, Danderyd, Sweden). Target xMAC (minimal alveolar concentration) was 0.4–0.6, resulting in an endtidal concentration between 0.5 and 1.3 Vol % depending on sex and age. Exhaled sevoflurane was measured with a “Vamos” monitor system (Drägerwerk AG & Co. KG, Lübeck, Germany). Volatiles are not yet licensed to sedate ICU patients; therefore, this indication is off-label use. When connecting an AnaConDa™ to Dräger Evita^®^ Infinity^®^ V500 respirator, it is mandatory to continuously measure the inspiratory and expiratory sevoflurane concentration. With this setting, inhalative sedation has proven to be safe for ICU sedation and is covered by national guidelines [31].

Sedation target during PP was a calm patient without pronounced or potentially harmful body movement concordant with preserved sufficient spontaneous breathing (pH > 7.2). Limited by the difficulty to accurately assess the Richmond Agitation and Sedation Scale (RASS) [32] in PP, this most likely corresponds to a RASS score of − 4 (“no response to voice, but movement or eye opening to physical stimulation”). When returning to supine positioning, sevoflurane was immediately discontinued aiming for a RASS Score of 0 for early mobilization and physiotherapy.

Patients also received basic analgesia with opioids, mainly gastric-retarded morphine or oxycodone. Especially during the start of the study period, patients received continuously infused sufentanil instead. Intravenous piritramid, a low-potent opioid, was additionally applied as nurse-controlled analgesia (NCA). As the visual analogue scale (VAS) cannot be applied in pronely positioned sedated patients, criteria for opioid application were symptoms of stress or pain like tachypnea, tachycardia, sweating, body motion, etc.

### Ventilator settings during prone positioning

Positive end expiratory pressure was set at between 12 and 16 mbar. Inspiratory fraction of oxygen (FiO_2_) was set aiming for an arterial oxygen partial pressure (paO_2_) of ≥ 60 mmHg. Patients were converted to the pressure-supported spontaneous breathing (PS) mode “SPN-CPAP/PS” (spontaneous-continuous positive airway pressure/pressure support) at the Evita^®^ Infinity^®^ V500 respirator (Drägerwerk AG & Co. KG, Lübeck, Germany). Criteria for reconverting to PCV were respiratory acidosis with blood pH < 7.2. Respirator settings were switched to the pressure-controlled ventilation (PCV) mode “PC-BIPAP” (pressure control biphasic positive airway pressure); opioid application was reduced and when patients started breathing spontaneously, the respirator again was switched to SPN-CPAP/PS mode. If spontaneous breathing could not be established promptly and accumulated opioids were the most likely reason, naloxone was administered in 0.04 mg steps in line with the responsible nurse’s discretion. Basic opioid therapy was then reduced. Nurses were advised to aim for tidal volumes of ≤ 8 ml/kg predicted body weight [2] (males = 50 + 0.91[height (cm)-152.4] kg; females = 45.5 + 0.91[height (cm)-152.4] kg) by modifying pressure support and endtidal sevoflurane concentration and applying opioids. The detailed standard operating procedure is illustrated in Fig. 1.

### Extracorporeal membrane oxygenation (ECMO)

High flow venovenous ECMO was indicated and implanted by two experienced intensivists when paO_2_/FiO_2_ fell below 60 despite PP or when respiratory acidosis was not controllable with lung-protective ventilator settings. Contraindications were lung failure not likely to resolve within a foreseeable period, prolonged ventilator dependence before referral, malignancies and severe underlying diseases. Details about ECMO assembly and thresholds have been published elsewhere [33, 34].

### Data collection

Detailed data about the ventilation mode, circulatory parameters, RASS and sevoflurane consumption were collected from the electronic chart (Copra 5, Copra Systems GmbH, Berlin, Germany). Reports on the patients’ condition were documented every 8th hour by both nurses and intensivists in the electronic chart. Every shift report was screened with special regard to unexpected events, especially harmful body motion, self-extubation or hemodynamic instability for this analysis.

### Statistics

Data were collected in Microsoft Excel sheets (Microsoft, Redmond, Washington, USA). Statistical analysis was performed with GraphPad InStat^®^ (GraphPad Software Inc, La Jolla, California, USA) to calculate the median and inter quartile range (IQR).

## Results

### Patients

**Fig. 2:**
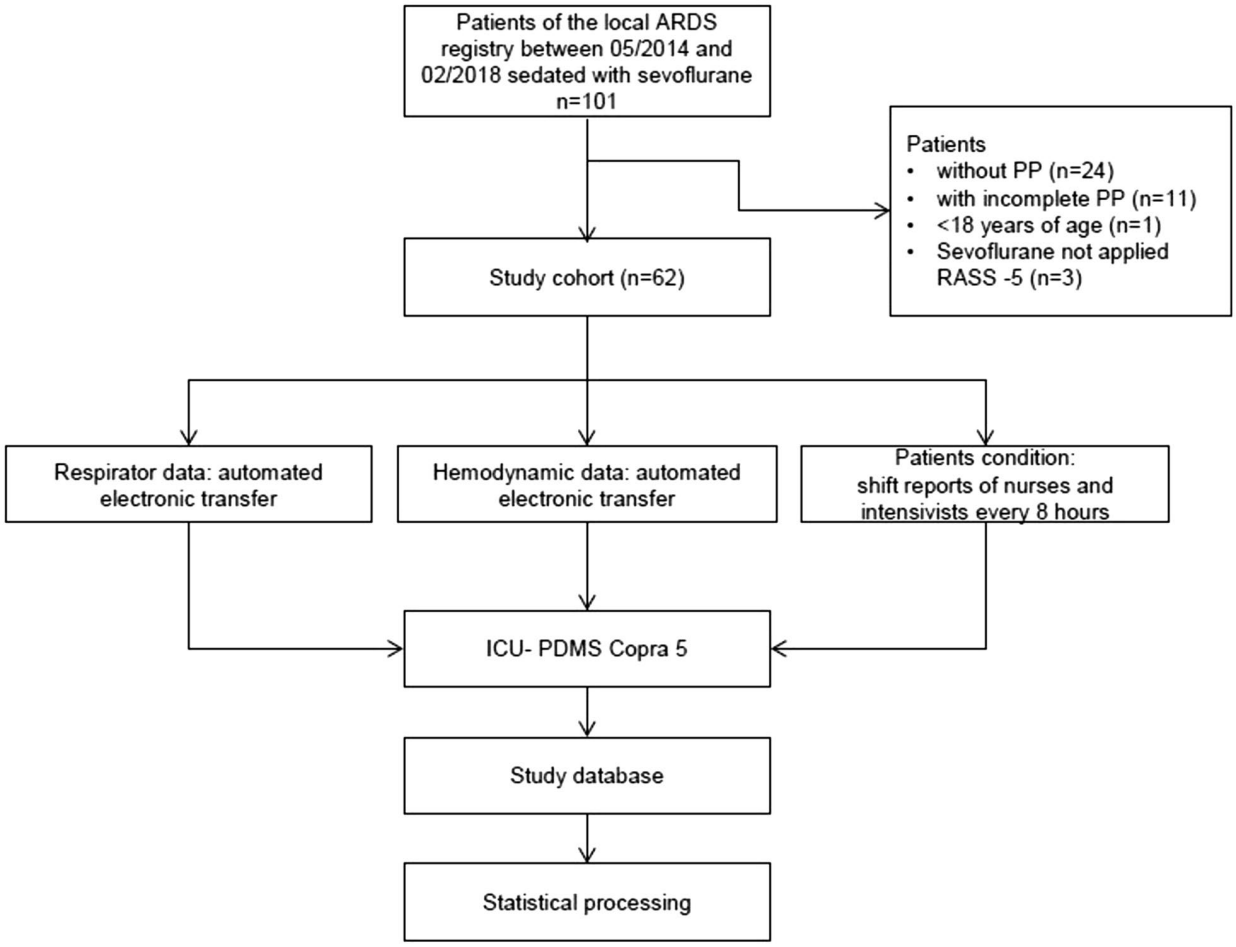
Selection of patients and data generation

For details on patient selection, please see Fig. 2.

Cohort characteristics are listed in Table 1.

**Table 1:** Patient characteristics (*n* = 62)

Patient characteristics	
Sex, *n* (%)	
Male	45 (73)
Female	17 (27)
Age [years], median (IQR)	56 (25.75)
Body mass index [m/l^2^], median (IQR)	25.4 (4.8)
Reason of ARDS, *n* (%)	
Bacterial lung infection	16 (26)
Aspirationpneumonitis/aspirationpneumonia	15 (24)
Influenza	13 (21)
ARDS secondary to extrapulmonary infection	7 (11)
Polytrauma	3 (5)
Others (post-lung surgery/transplant, etc.)	8 (13)
ICU Mortality [%]	47
ICU stay [days], median, (IQR)	15 (14)
PaO_2_/FiO_2_ at ICU admission/before ECMO implantation by ECMO outreach team, *n* (%)	
201–300	2 (3)
101–200	26 (42)
≤ 100	34 (55)
TISS-10, median, (IQR)	20 (12)
SAPS II, median, (IQR)	48 (17.25)

*TISS* Therapeutic Intervention Scoring System [35], *SAPS* Simplified Acute Physiology Score [36]

Three patients in PP already presented RASS values of − 5; thus, prescribed sevoflurane was not applied in line with the responsible nurse’s discretion. Two patients with only “mild” ARDS had been intubated before for other reasons and were brought to PP because respiratory function would probably worsen in due course.

### Primary endpoint: duration of pressure-supported spontaneous breathing

**Fig. 3:**
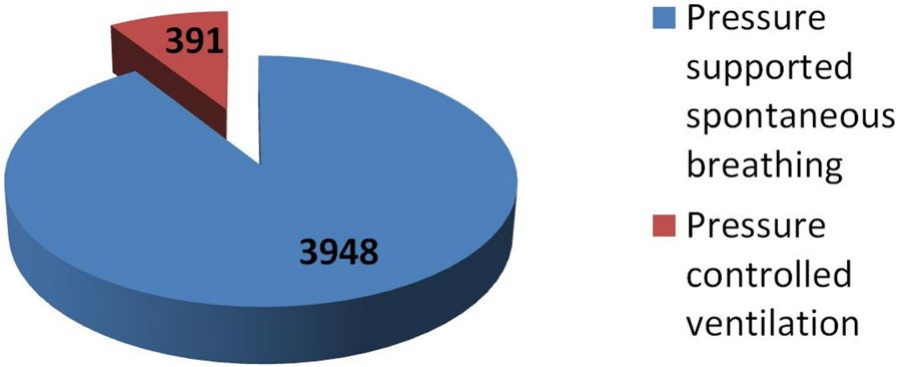
Respirator mode during prone positioning (cumulative hours)

Overall, 4339 h of prone positioning have been observed. Within 3948 h (91%), patients were switched successfully to a pressure-supported spontaneous breathing mode (Fig. 3*)*.

The median duration of each prone episode was 17 h (IQR 3). Median duration of pressure-supported spontaneous breathing per episode was 16 h (IQR 5). Results are listed in Table 2.

**Table 2:** Details on episodes of prone positioning

Episodes of prone positioning	
Overall, *n*	276
Per patient, median, (IQR)	4 (4)
With ECMO	152
Without ECMO	124
Sevoflurane consumption/episode [ml], median (IQR)	48 (17)
Duration [h], median (IQR)	17 (3)
Cumulative hours of PP	4339
Cumulative hours of PS during PP	3948
RASS, median (IQR) during PP	− 4.5 (1.5)
pH before conversion to PS, median (IQR)	
Prone episode 1	7.313 (0.116)
Prone episode 2	7.35 (0.102)
Prone episode 3	7.356 (0.103)
Prone episode 4	7.374 (0.117)
Prone episode 5	7.371 (0.092)

*PP* Prone positioning, *PS* pressure-supported spontaneous breathing; *RASS* Richmond Agitation and Sedation Scale [37]

### Reasons for failed conversion to pressure-supported spontaneous breathing

As presented in Fig. 1, conversion to PS was intended when safe preconditions had been established. If pH was < 7.2, patients were reconverted to PCV. We identified 2 factors for failed PS attempts: Some patients suffered from accumulation of opioids or sedatives they had received in the referring hospitals before. Despite treatment with naloxone, PS could not be established promptly. The other cohort failed breathing spontaneously were ECMO patients with very low tidal volumes (< 100–150 ml). In these patients, alveolar concentration of sevoflurane was difficult to set and obviously too low to satisfactorily suppress agitation.

### Complications and unexpected events

Unexpected events related to prone positioning or inhaled sedation are listed in Table 3.

**Table 3:** Complications and unexpected events related to prone positioning or inhaled sedation

Patient number	Type of complication	Reason	Management	Result
063	Pharyngeal dislocation of endotracheal tube	Unclear whether the patient extubated herself with her tongue or fixation of the tube unsticked because of salivation	Already spontaneous breathing → Noninvasive Ventilation, turned back, re-intubation	No hypoxia, turned back in PP after re-intubation
019	Insufficient sedation	Technical defect of Anaconda	Dexmedetomidine infusion	PP continued
094	Insufficient sedation	Low tidal volume	Sufentanil infusion	PP continued
095	Insufficient sedation	Low tidal volume	Sufentanil infusion	PP continued
056	Respiratory acidosis	Unclear	Controlled ventilation → still acidodic → turned back to supine position, Anaconda removed	Still acidotic in supine position
072	Respiratory acidosis	Retrospectively: kinked tube	Turned back, refixation of tube	PP continued

*PP* Prone positioning

Three circulatory arrests have been observed in temporary but not clearly in causal connection with prone positioning and sevoflurane-sedation:

One patient (number 051) suffered from septic shock with abdominal focus. Because of multiorgan failure und underlying morbidities, this patient had been considered ECMO-unsuitable. He was turned to prone positioning as ultima ratio because of severe hypoxia. Despite mildly improved oxygenation, he died of uncontrolled lactic acidosis.

One patient (number 097) connected to venovenous ECMO developed severe septic shock and acute renal failure following pneumonia. Due to metabolic acidosis and increased serum potassium, he presented with ventricular tachycardia and severe hypotension and was successfully resuscitated after turning to supine position but died from septic shock in the following hours.

One patient (number 101) developed right heart failure. He was turned back and resuscitation was started. After returning to spontaneous circulation continuously infused epinephrine and inhaled nitric oxide were started. Unfortunately, the patient died the next day.

## Discussion

This observational feasibility trial is the first to systemically evaluate inhaled sedation with sevoflurane for PP in combination with PS. Conversion to PS succeeded in 91% of the cumulative hours of PP. During 276 episodes with over 4000 h of PP, no related fatal event occurred. With a median RASS value of − 4.5, a sufficient sedation depth was achieved in 59 (95%) out of 62 patients with a combination of sevoflurane and opioids.

The finding that PS during PP in intubated ARDS patients is safely achievable during volatile sedation with sevoflurane forms the basis from which to question the current dogma in ARDS treatment. On the other hand, this approach inherits the risk of hyperinflation due to increased respiratory effort. This concept must therefore be investigated in comparison with controlled ventilation in terms of driving pressure, lung-protective parameters, muscle weakness and mortality before it can be routinely applied.

### Tolerating Prone Positioning and ventilation versus avoidance of long lasting sedatives

There is still no convincing evidence for what constitutes the optimal respirator mode to achieve lung-protective parameters and to decrease driving pressure in ARDS [17]. In the 2010 “PROSEVA” - Trial authors compared controlled ventilation and controlled ventilation in addition with muscle relaxation in ARDS. Patients revealed significantly better survival when they additionally received the muscle relaxant cis-atracurium in early ARDS [38]. Anti-inflammatory effects via improved patient-ventilator synchrony have been discussed in conjunction with this finding. Deep sedation is recommended when muscle relaxants are used. Patients in the “PROSEVA” Trial in both groups were deeply sedated to a Ramsey Score of 6 (“patient exhibits no response”). In contrast to this approach, intensivists have become aware that deep sedation and ICU-acquired muscle weakness lower survival and quality of life after hospitalization [39, 40]. A relevant side effect of prolonged sedation is a loss of muscle strength [23]. Current guidelines therefore ask for conscious patients who can interact and actively participate during their healing process [15, 31, 41, 42]. Inhaled sevoflurane [43] allows a compromise: Sufficient sedation during PP and rapid awakening [44] help enable active physiotherapy and mobilization during episodes of supine positioning.

Crotti et al. [45] found that spontaneous breathing in ARDS during ECMO was feasible in only 30% of her patients. All of them received intravenous sedative drugs. Pressure-supported spontaneous breathing was achieved in 95% of our patients for more than 90% of the cumulative time in prone positioning. In our opinion, this is an effect of the beneficial properties of sevoflurane: Mild and moderate concentrations below depressive effects to the respiratory genesis can easily be set compared to intravenous sedatives.

### Advantages of spontaneous breathing

There are various possible advantages of PS during ARDS: Muscle activation may help prevent diaphragm atrophy [14] and active inspiration may help distribute air within the lung more evenly [46, 47] and in areas with low ventilation/perfusion ratios. Spontaneous breathing has the potential to lower intrathoracic pressure and therefore contribute preventing right heart failure—a highly relevant comorbidity contributing to ARDS mortality. In addition to hypoxic pulmonary arterial vasoconstriction, increased intrathoracic pressure inherits the risk of impairing right ventricular function by hindering venous blood’s return to the right atrium and increasing right ventricular afterload [12, 16, 48].

The main concern associated with spontaneous breathing during ARDS is the increase in transpulmonary pressure with consecutive spatial hyperinflation. This effect seems to mainly threaten the ventral areas of the lung during supine position. Stabilizing the chest wall’s anterior through the patient’s bodyweight in PP alleviates this theoretical harm during PS [5, 18, 49].

### Respiratory drive and respiratory genesis

The genesis of respiration and the respiratory drive are controlled by complex feedback loops and dependent on various parameters (paO_2_, paCO_2_, pH, inflammation, body temperature, agitation, etc.) described elsewhere [50, 51]. Target values for paO2 [52] and blood pH [53, 54] have been discussed especially. While better oxygenation obviously reduces inspiratory effort and the respiratory rate, it is difficult to achieve in severe ARDS. It is even more difficult to define an evidence-based threshold for blood pH before converting patients to PS. Crotti for example included 30 patients with ARDS in her spontaneous breathing study [45]. She did not find a significant difference between blood pH values in the 22 patients in whom spontaneous breathing was unfeasible and eight patients with spontaneous breathing. In our workup flowchart (Fig. 1*),* we chose arbitrary thresholds of > 60 mmHg for paO2 and pH > 7.2, respectively, to establish FiO_2_ and pressure support. The analgosedation regimen based mainly on varying the endtidal sevoflurane concentration and opioid application does not specifically address all the aforementioned parameters. During over 90% of cumulative hours of PS during PP, however, it really worked in clinical practice even in patients with blood pH values below 7.2.

### Effects of inhaled anesthetics

Many side effects of volatile anesthetics have been reported recently [26, 55–57] which may partly be advantageous in ARDS: Sedation with sevoflurane improves oxygenation and decreases levels of inflammatory markers in ARDS compared to midazolam [57]. Bronchodilatory, anti-inflammatory [58] and even anti-bacterial properties [59, 60] have been reported. Cardio-protective effects also have been discussed [61].

### Additional effects

Some patients in this study were able to turn to PP themselves with assistance. This advantage is chosen, when patients are alert and calm while in supine position and when physiotherapists confirm sufficient muscle strength. Lines and tubes are secured by the nurse and an intensivist at the head-end of the bed. Patients are instructed beforehand and then allowed to turn around and lay themselves in prone positioning until they feel comfortable. They are able to communicate with their hands and by the help of a mirror reflecting the face. Sevoflurane is not initiated until patients have clearly agreed. We believe that this innovation may help us lower the incidence of skin bruises and positional damage.

### Severity of ARDS and mortality

Mortality was not an endpoint of this study. Most of the patients at ICU admission had severe (55%) or moderate (42%) ARDS. Two intubated patients were admitted to ICU with “mild” ARDS (Table 1*)*. One of them (No 67) was put into PP because pulmonary function was rapidly worsening and severe ARDS developed in due course. The other patient (No 78) was extubated after the first PP episode, and treatment was continued with noninvasive ventilation. Ten patients had been diagnosed with severe ARDS in primary hospitals and were then connected to ECMO by our outreach team and referred to our ICU. The 47% mortality in our cohort is therefore comparable or even lower than expected when compared to epidemiological data [62].

### Limitations

This “proof-of-principle” trial’s main drawback is its retrospective design. A control group would have been desirable. The use of inhaled anesthetics requires deep understanding of malignant hyperthermia, and dantrolene must be at hand. While absorbers and gas exhaustion systems keep the ambient air load minimal, the global warming potential of sevoflurane is enormous [63]. The Anaconda and the Anaconda-S device increase the dead space by 100 ml and 50 ml, respectively, which can increase respiratory acidosis and alveolar concentration of sevoflurane especially in low tidal volumes [64, 65].

## Conclusions

Pressure-supported spontaneous breathing in patients undergoing prone positioning in ARDS is feasible and safe during inhaled sedation with sevoflurane—a concept that challenges current state-of-the-art ARDS therapy. Research should now focus on comparing controlled and pressure-supported spontaneous ventilator settings during PP and the effects on driving pressure, lung-protective parameters, muscle weakness and mortality in a larger cohort. These investigations are essential before the PS approach during PP can be routinely recommended.

## Abbreviations

ARDS: acute respiratory distress syndrome
BIPAP: biphasic positive airway pressure
CPAP: continuous positive airway pressure
ECMO: extracorporeal membrane oxygenation
ICU: intensive care unit
IQR: interquartile range
PP: (Prolonged) prone positioning
PS: pressure-supported (spontaneous breathing)
RASS: Richmond Agitation and Sedation Scale
SAPS: Simplified Acute Physiology Score
TISS: Therapeutic Intervention Scoring System
